# A Review of Photon-Counting Computed Tomography (PCCT) in the Diagnosis of Cardiovascular Diseases

**DOI:** 10.7759/cureus.73119

**Published:** 2024-11-06

**Authors:** Astha Sharma, Maria Gabriela Cerdas, Setareh Reza-Soltani, Vikash Rustagi, Manojna Guntipalli, Diana Stefanie Rojas Torres, Mrinal Bhandari, Shreya Kandel, Dharma Teja Rayaprolu, Mohammed Hussain

**Affiliations:** 1 Medicine, University Hospital Birmingham NHS Foundation Trust, Birmingham, GBR; 2 Medicine, University of Medical Sciences (UCIMED), San Jose, CRI; 3 Radiology, Advanced Diagnostic &amp; Interventional Radiology Center (ADIR) Tehran University of Medical Sciences, Tehran, IRN; 4 Radiology, Istituto di Ricovero e Cura a Carattere Scientifico (IRCCS) Ospedale Galeazzi, Milano, ITA; 5 Medicine, Gandhi Institute of Technology and Management (GITAM) Institute of Medical Sciences and Research, Visakhapatnam, IND; 6 Cadiology, Lundquist Institute for Biomedical Innovation at Harbor-UCLA Medical Center, Torrance, USA; 7 Cardiology, Lundquist Institute for Biomedical Innovation at Harbor-UCLA Medical Center, Torrance, USA; 8 Medicine, University Hospital Southampton NHS Foundation Trust, Southhampton, GBR; 9 Internal Medicine, Mamata Medical College, Khammam, IND; 10 Respiratory Medicine, Royal Stoke University Hospital, University Hospitals of North Midlands NHS Trust, Stoke-on-Trent, GBR

**Keywords:** cardiovascular imaging, contrast sensitivity, diagnosis, energy-integrating detectors, photon-counting computed tomography, spatial resolution

## Abstract

Photon-counting computed tomography (PCCT) is an innovative mechanism used for imaging and provides higher spatial resolution and contrast sensitivity in comparison with the orthodox energy-integrating detectors (EIDs). Unlike EID-based CT systems, which indirectly convert X-ray photons to electrical signals, PCCT directly counts and quantifies each photon’s energy, enhancing image quality and material separation. With all of these features, PCCT is especially useful for cardiovascular imaging, where it is essential to precisely observe cardiac tissues, vascular structures, and coronary arteries. Around the globe, cardiovascular diseases (CVDs) continue to be the primary cause of morbidity and death, and early, precise diagnosis is essential for effective management. This review examines the role of PCCT in diagnosing CVDs, highlighting its enhanced capabilities in improving the precision in diagnosis as well as patient outcomes compared to conventional CT methods. While current evidence supports PCCT's advantages, further research is necessary to validate these findings and facilitate its broader clinical adoption.

## Introduction and background

Recent years have seen major technological advancements in non-invasive cardiac imaging. As far as anatomical and angiographical evaluations in cardiovascular medicine are concerned, computed tomography (CT) is considered to be the fundamental tool for diagnosis. The necessity to examine coronary atherosclerotic plaque morphology, assess myocardial tissue functionality, and confirm or rule out substantial coronary luminal stenosis or abnormalities triggers the growing need for diagnostic imaging technology [1]. However, CT does have some limitations, such as limited contrast resolution, challenges while assessing the coronary lumen precisely when intensive plaque calcifications or small stents are present, and difficulties in inspecting components of plaque. Appearance of artifacts during conventional imaging process can result due to the severe calcification that would lead to deficient or false-positive results regarding stenosis [1,2]. Additionally, CT scanning also carries the risks of ionizing radiation exposure, contrast-induced reactions, and nephropathy [1].

Photon-counting computed tomography (PCCT) is a cutting-edge technology in imaging that outperforms CT methods. X-ray photons are converted into signals via photon-counting detectors (PCDs). It offers superior spatial resolution, along with minimally produced electronic noise, and the capability to capture multi-energy spectral data without prior selection [3]. These advancements greatly enhance capabilities. Recent technological progress has been geared towards enhancing resolution and reducing noise characteristics to improve image quality and minimize artifacts in low-dose scans. These advancements could completely transform cardiac imaging results by enabling evaluations of cardiac tissue, myocardial perfusion, coronary flow kinetics, and cardiac valve function [3]. The dual-source configuration of PCCT allows for acquiring high-resolution images, which are crucial for diagnosing cardiac conditions and evaluating coronary artery disease. This setup enhances accuracy by providing anatomical details in clinical environments [1]. By capturing X-ray photons at energy thresholds, PCCT boosts precision for detecting subtle cardiovascular abnormalities, providing a reassuring level of diagnostic capability [2]. PCCT marks an advancement in testing, providing better spatial detail, less radiation exposure, and improved diagnostic precision compared to conventional CT scanners. These progressions are on the verge of transforming the way clinical decisions are made in heart health by providing an understanding of structure analysis of plaque buildup and characteristics of heart tissue [4].

After 15 years of research and development, PCCT, an imaging mechanism attributed to energy-resolving, direct-conversion X-ray detectors, has recently been integrated into clinical CT equipment [5]. Depending on indirect X-ray modification (with scintillators) and signal aggregation across the entirety of the X-ray energy spectrum, this differs considerably from traditional CT detectors. PCCT is capable of transforming the clinical CT situation owing to its many integral benefits and capacity to address a number of existing state-of-the-art CT systems' inadequacies [5].

The intent of this narrative review is to provide insight into the PCCT's computational regulations, its applicability under the category of cardiac imaging, and its effectiveness in cardiovascular diagnostics, as well as an end-note discussion of its future directions and advancements.

## Review

Technical principles of PCCT

**Figure 1:**
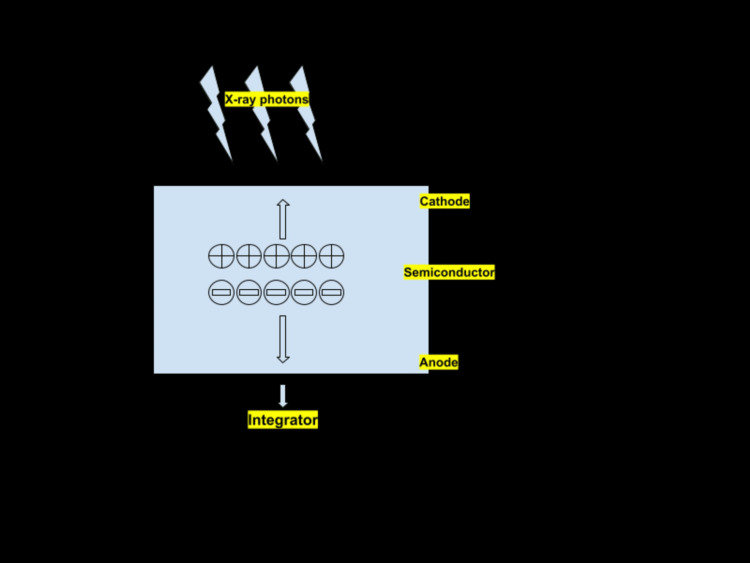
Schematic representation of PCCT PCCT, photon-counting computed tomography

PCCT is a cutting-edge imaging technique that provides significant advantages over conventional CT in terms of noise reduction, reduced artifacts, better image resolution, and improved contrast between different tissues [5]. PCCT works by detecting the photons that are released during an X-ray and converting them directly into an electrical signal. The conventional CT uses energy-integrating detectors (EIDs), which convert the photons of the incoming X-rays into light energy [5]. This energy is further detected by photodiodes made of semiconductor materials, which convert the energy into electrical signals, which are further converted to digital signals. The cumulative efficiency of all the X-ray photons is indicated by the perceived signal. Consequently, the traditional CT scan indirectly detects the X-ray photons' energy. PCCT works on the principle of direct conversion. PCCTs use semiconductor materials such as cadmium telluride and silicon. The semiconductor is embedded between a pixelated anode and a cathode [6]. The amount of energy of the incoming photons promptly produces electron-hole pairs. The height of the electric pulses generated with the charges is precisely equivalent to the energy of a single X-ray photon. A comparative analysis of all the pulses is performed, and the photons are grouped into energy groups or bins; therefore, the photons are differentiated based on their energy levels [7] (Figure 1).

Benefits of PCCT in cardiovascular imaging

Advanced imaging methods such as PCCT have proved extremely beneficial in the detection and management of cardiovascular disorders. This includes an improved image resolution, reduced radiation dose, and better tissue characterization [8,9]. PCCT provides a superior spatial resolution compared to traditional CT by allowing detailed visualization of small cardiovascular structures [10]. It improves contrast resolution and helps differentiate between tissue types and pathological changes [5,11]. In current EIDs, the image resolution is limited due to their pixel size (0.4-0.6 mm at the isocenter). However, when using PCDs, the pixel pitch does not have a mechanical separation and therefore no technical limitation, allowing a smaller pixel size (0.15-0.225 mm at the isocenter), leading to enhanced spatial resolution [5,9,12].

A great benefit of PCCT is its capacity to achieve comparable image quality with a reduced radiation dose. PCCT offers an enhanced dose efficiency compared to EID due to its superior contrast-to-noise ratio (CNR), reduced electronic noise, and improved capacity to perceive tiny details. Patients in clinical practice are going to take advantage of this since it promotes less radiation exposure without compromising image quality [2,4]. Unlike EID, PCD reduces electronic noise and facilitates optimal photon energy weighting, which can be used to decrease the doses of radiation [10]. Meloni et al., when comparing PCD to EID, concluded that the former showed images with fewer streak artifacts and reduced noise, enhancing dose efficiency [5]. In clinical settings, the ultra-high spatial resolution and artifact reduction provided by PCCT could greatly enhance patient care by producing detailed cardiovascular imaging [13].

Comparison of PCCT with other imaging modalities

PCCT is a revolutionary breakthrough in imaging technology that is advantageous over CT scans, MRIs, and echocardiograms in certain medical situations [13]. PCCT stands out for these reasons. Firstly, it excels in generating high-resolution images, with clarity ranging from 0.15 to 0.225 mm, thus surpassing CT scans and producing sharper, more detailed visuals while reducing partial volume effects. In contrast to CT scans, PCCT employs PCDs to significantly improve the CNR and diminish noise, which improves picture clarity and makes low-attenuation materials easier to see [14]. This capability is particularly valuable for evaluating stenoses and stent lumens. Furthermore, the multi-energy data acquisition feature of PCCT allows for material differentiation distinguishing contrast materials and enhancing analysis [5]. This functionality plays a role in imaging research by offering insights into tissue properties and pathology assessment. Additionally, PCCT minimizes artifacts such as beam hardening effects and metal artifacts through enhanced resolution and improved material differentiation processes, thereby improving accuracy. In applications, PCCT demonstrates potential compared to MRI scans and echocardiograms [4,15].

While MRI is effective in distinguishing between tissues, PCCT stands out for its ability to produce images without the need for contrast agents and conduct energy scans, enhancing tissue analysis without concerns about radiation exposure [16]. In comparison to echocardiography, which struggles with operator dependence and acquiring views, PCCT delivers anatomical and functional information ensuring dependable clinical evaluations [17]. Despite being in its stages, PCCT shows promise in revolutionizing imaging by leveraging the strengths of CT, MRI, and echocardiography while addressing their limitations. With its resolution, reduced radiation exposure, and capability to minimize artifacts, PCCT proves to be an imaging technique suitable for various clinical settings, offering unmatched diagnostic precision and patient safety [18]. As advancements progress further, PCCT could emerge as a tool in imaging practices by establishing benchmarks in cardiovascular healthcare and beyond.

PCCT in clinical practice

PCDs could split the initial X-ray energy spectrum into multiple energy bands. Enhanced spatial and contrast resolution, minimized radiation exposure, lesser image noise and artifacts, and multi-energy imaging based on the atomic properties of tissues, which facilitates the use of multiple contrast agents and enhances quantitative imaging, are among the positive attributes of PCCT over conventional CT technology [5].

Soschynski et al. found PCCT to be highly effective in assessing the coronary artery by providing high-quality images that allowed good accessibility of coronary segments and vessels even in cases of calcified plaques and stents [19]. According to Symons et al., in ICA portions near the surrounding bone, PCCT representations displayed less severe beam hardening artifacts and 9% reduced image noise, hence proving its potentially beneficial application in brain and vascular imaging to improve image quality [20]. Sartoretti et al. concluded that PCCT along with tungsten as contrast media improved the coronary artery imaging by enhancing the imaging of lumen and plaque vascularization more competently [21]. Leng et al. concluded that PCCT provided superior morphological and compositional imaging of renal stones compared to standard CT, leading to better visualization of the stones’ features and nature [22].

Baffour et al. reported that Ultra-high-resolution PCCT imaging improved visualization of the pelvic and shoulder osseous structures while using 31-47% less radiation [23]. Symons et al. concluded that lung cancer screening at lower doses with PCCT showed better Hounsfield units for various tissues and exhibited less noise and a higher CNR compared to conventional EID [24]. Pourmorteza et al. demonstrated that a standard PCCT for brain imaging shows improved image quality compared to EID-CT, with 12.8-20.6% reduction in image noise and a 15.7-33.3% increase in soft-tissue CNR [25].

In humans, coronary CT angiography (CCTA) by means of a PCCT system demonstrated enhanced image clarity and diagnostic confidence when compared to an energy-integrating dual-layer CT [10]. From vascular and plaque imaging, renal stones, osseous structures visualization, screening of lung cancer, and improved brain imaging, PCCT has proven to be a better adaptive technological advancement in the radiological field.

PCCT provides several advantages over conventional CT. Koons et al. found that PCCT systems achieved excellent spatial resolution and substantial reductions in noise (up to 47%) and dose (up to 30%) compared to conventional detector-equipped systems [8,9,25]. Numerous studies have assessed PCCT's effectiveness in the detection of cardiovascular conditions in recent years.

The ideal non-invasive approach for analyzing coronary artery disease is CCTA [13,26]. However, traditional CCTA offers restricted soft tissue contrast and spatial resolution. PCCT allows high-resolution imaging of coronary arteries, leading to superior image quality [12]. A study by Si-Mohamed et al. reported that PCCT images showed elevated detectability indices for the coronary lumen and non-calcified plaques (2.3 and 2.9 times greater respectively) compared to conventional CT images [8].

A study by Mergen et al. concluded that PCCT enhanced the quantitative characterization of coronary plaque, minimizing blooming artifacts and strengthening the detection of the lipid-rich and fibrotic plaque constituents [27]. In addition, Rotzinger et al. found that in terms of determining the lipid-rich, simulated non-calcified plaques in coronary arteries, spectral PCCT performed more effectively than classical EID-CT [28]. Furthermore, a study by Soschynski et al. concluded that PCD CCTA provided excellent image quality and a high CNR, ensuring that 95% of coronary segments were accessible [19]. Hagar et. al conducted a prospective study in which patients with intense aortic valve stenosis were evaluated with the help of PCCT angiography. The study achieved a sensitivity of 96%, specificity of 84%, and accuracy of 88%, proving a high diagnostic precision in individuals who were at a higher risk for coronary artery disease [26].

Quantification of coronary artery calcium (CAC) has been employed as a CAD screening approach and has established predictive value for cardiovascular disease. On the other hand, it is well recognized that the diagnostic accuracy of CCTA is negatively impacted by the presence of highly calcified plaque in the coronary arteries [9]. For this reason, Allmendinger et al. evaluated a new image reconstruction algorithm (PureLumen) based on spectral CT data from dual-source PCT-CT. It indicated that it might improve the interpretability of images by reducing blooming artifacts from calcified plaques [29].

CT has been used to evaluate ischemia and scarring to assess the degree of damage to the myocardium. The myocardial extracellular volume (ECV) is increased in most conditions that impact the heart muscle [30-39] Mergen et al. conducted a study to explore the viability and precision of myocardial ECV using cardiac PCCT. The study reported that this technique, which used a late enhancement cardiac dual-energy scan at a low radiation dosage, permitted accurate ECV measurement [27].

PCCT is an innovative technique of imaging, which appears to have a lot of potential. With PCCT, it’s possible to reduce radiation exposure while simultaneously improving image resolution, quality, and iodine CNR-achievements that are nearly impossible with conventional modalities [30]. When compared to EID, PCCT exhibits lower noise, improved CNR by around 29-41%, and an improvement in detectability index of 20-36% [31].

In cardiology, where visualization of coronary arteries, cardiac tissues, and associated pathologies should be performed with accuracy, PCCT has shown enhanced results in the detection and characterization of coronary artery disease while differentiating calcified plaques in those arteries from iodinated contrast material [32].

Current evidence reveals that PCCT devices have a higher table load capacity than conventional scanners, highlighting their use in patients with obesity [14]. In children, the advantages are numerous when using PCCT, as this led to a major decrease in cumulative radiation exposure when compared with traditional CT scanners [14].

With the advancement of PCCT and its role in cardiac imaging, there are still limitations associated with it. A significant obstacle lies in the requirement for additional research and verification of optimizing parameters and reconstruction for different clinical situations because of new and developing technology [1]. Moreover, while PCCT has shown promise in lowering the artifacts and providing more accurate assessments of coronary stenosis and plaque characteristics, availability is an issue, and its clinical adoption is limited by the need for more extensive validation studies and approval processes [1].

Insufficient focal spot size can lead to degradation of overall image quality. To fully use the benefits of dose reduction and improved image quality, there is a requirement of a wider energy range for spectral characteristics of PCCT [11].

Few important technical challenges are seen from the conflicting factors. These challenges can be divided into photon flux independent and photon flux dependent effects. The effects that are independent of photon flux include charge sharing, charge trapping, and K fluorescence escape, whereas pulse pileup falls in the category of photon flux dependent effects. The high cost, big data files, data storage, and post-processing of PCCT are other big challenges in current clinical practice [1].

Specific applications of PCCT in different cardiovascular diseases

PCCT represents noteworthy progress in cardiac imaging, offering detailed myocardial characterization and enhanced diagnostic capabilities for various cardiomyopathies. Its ability to provide high-resolution images, distinguish between different tissue types, and assess myocardial perfusion make it a powerful tool in the evaluation and management of heart diseases [1,2].

PCCT will be crucial in visualizing heart muscles. In particular, it is anticipated that PCCT would enhance the measurement of cardiac calcium, myocardial ECV, myocardial radiomic characteristics, and EAT and PCAT (epicardial and pericoronary adipose tissue). The present study investigates the function of PCCT in cardiac and myocardial characterization in cardiovascular imaging. Furthermore, PCCT may improve the qualitative investigation of coronary plaques and stents [33]. PCCT’s ability to perform spectral imaging allows for better differentiation of materials based on their atomic numbers, which is useful for identifying specific tissue types and compositions. This is particularly important in cardiac imaging for distinguishing between different types of myocardial tissue and detecting fibrosis [34].

During a CCTA, late-enhanced CT images are taken 5-10 minutes after injecting iodinated contrast. The enhanced areas in these scans are mainly due to myocardial fibrosis from conditions such as myocardial infarction, amyloidosis, myocarditis, and cardiomyopathies. To measure the extracellular matrix in the myocardium, the ECV fraction can be calculated. This is done by comparing pre-contrast and post-contrast images, adjusting for iodine changes in the blood pool. Although PCCT offers better accuracy by reducing artifacts, it is not yet widely available as it requires further validation and approval [12].

CT can be used to carry out the non-invasive estimation of myocardial fibrosis. For patients who are unfit for MRI, myocardial ECV quantification along with CT is considered a substitute. The measurement of cardiac ECV using CT and ECV using MRI exhibit an excellent correlation [12,35]. For a variety of heart-related diseases, such as cardiac amyloidosis, incremental predictive information is provided by myocardial ECV measurement, and, on the other hand, CT is less prone to artifacts and allows for a quicker acquisition time for ECV assessment. Furthermore, compared to MRI, CT scanners are easier to access and provide a more rapid, inexpensive, non-invasive technique for assessing ECV [18].

In a study by Aquino et al., ECV CT was calculated in a total of 29 patients (13 of whom had known cardiomyopathy 4 of whom had prior myocardial infarction) using both the single-energy and dual-energy approach, and these values were then compared with ECV MRI while using a first-generation dual-source PCCT [17]. Significant association was observed among dual- and single-energy-based techniques (r=0.91, p<0.001); with the dual-energy approach, the likelihood of radiation reduction appeared to be 40% by dosage. Other than that, for both global and midventricular quantification, there was a significant correlation between ECV MRI and ECV CT as identified by dual-energy PCCT (r=0.82 and 0.91, both p<0.001) [17]. Assuming that PCCT is readily accessible, ECV CT calculations may be performed using less contrast medium and radiation [36].

PCCT represents a significant advancement in cardiac imaging, particularly for diagnosing and managing cardiomyopathies. Its high-resolution imaging, ability to precisely quantify myocardial ECV, and reduced radiation exposure position PCCT as a revolutionary tool in the cardiovascular field, enhancing both diagnostic accuracy and patient safety [36]. PCCT offers superior cardiovascular imaging compared to conventional CT. It improves the accuracy of coronary plaque analysis, luminal stenosis, and calcium quantification, even at lower radiation doses. PCCT also provides detailed insights into plaque composition, biological activity, and stent evaluation, while reducing the required iodine contrast without compromising diagnostic quality [4].

PCCT has become a groundbreaking tool for detecting and managing coronary artery disease due to its advanced imaging resolution and the dynamic, multi-energy data it provides [1]. Higher image resolution is a proficient tool in visualizing smaller anatomical structures such as coronary lumen, stent placement [1], and plaque characteristics such as quantification, calcification, and progression [3]. PCCT allows for lower radiation doses, higher iodine contrast, and lower contrast volumes. Koons et al. reported that PCCT provided a more accurate assessment of coronary lumen stenosis. PCCT can detect iodine in the lumen of the coronaries having plaques with greater than 75% stenosis, which were previously considered impervious due to the lumen being too narrow [9]. Rotzinger et al demonstrated that although it involved detecting non-calcified, rich lipids plaque in coronary arteries at lower radiation doses, PCCT outperformed standard CT [28]. The effect was more prominent in large-sized patients who are frequently exposed to higher radiation doses to achieve comparable images [28]. Si-Mohamed et al. through an in vivo study involving 14 patients reported that PCCT leads to improvement in presentation in regard to characteristics of calcification, stent, and non-calcified plaque by 100%, 92% (95% CI: 71, 98), and 45% (95% CI: 28, 63), respectively [8].

Traditional imaging faces the challenges of blooming artifacts due to severe calcification in the coronaries, thereby decreasing the image quality. Li et al. confirmed that PCCT improved the accuracy of stenosis measurement [37]. Errors in imaging were significantly more using single-energy computed tomography, i.e., conventional CT, than multi-energy computed tomography, i.e., PCCT [37].

CAC score is considered an accurate predictor of cardiovascular disease burden [37]. PCCT has improved the accuracy of CAC score quantification by reducing blooming artifacts, which leads to overestimation of the CAC score. It also prevents underestimation by measuring the thinner calcification, which is often missed by the conventional CT technique [2]. Although CAC is considered an important predictor of cardiovascular outcomes, it is not an indicator of mortality, which directly correlates to plaque progression and rupture. Rotzinger et al. reported the efficiency of PCCT in identifying lipid core and non-calcified atherosclerotic plaque [28].

Coronary artery stent placement is popularly used to treat stenosis, helping in curing symptoms, improving the life quality of the patient, and lowering the chance of future complications related to heart [4]. On CT imaging, metal stents present thicker because of blooming artifacts and lead to restrict evaluation in-stent lumen. In addition, the close spacing of the stent struts also makes it challenging to see them clearly [38]. On the other hand, in-stent restenosis (ISR) is a common problem with this treatment, which occurs more frequently with bare-metal stents compared to drug-eluting stents. CCTA has proven to be highly reliable in ruling out significant ISR after stent implantation. However, challenges remain in visualizing certain stents due to factors such as limited spatial resolution, beam hardening artifacts, blooming, and metallic interference [4]. Various lab studies have assessed the potential of PCCT to address these restrictions.

Stein et al. conducted a thorough examination of how PCCT technology affects the assessment of small vessel stents compared to EID-CT [39]. They evaluated 12 water-contrast-filled stents of different sizes using PCCT and EID-CT with a patient-equivalent phantom. After reconstructing the images with vascular kernels, five radiologists rated image quality on a 5-point scale, with PCCT receiving the highest scores. PCCT showed significantly better sharpness and reduced blooming compared to EID-CT. The study concluded that PCCT, with a sharp vascular kernel, offers the best image resolution for small stent imaging, reducing the need for invasive coronary angiograms [39]. Similarly, Petritsch et al. compared the visibility and image quality of different coronary stents of PCCT system to a traditional (EID) CT with a phantom study [40]. The results showed that the PCCT system had superior visualization of the stent lumen and lower levels of image noise compared to the EID-CT system. The differences between the two systems appeared significant statistically, and the PCCT system provides superior visibility and image quality for coronary stents [8]. PCCT may improve the detection and treatment of coronary artery disease [8,38].

CT scans and CT angiography (CTA) are commonly used before and after procedures such as EVAR and TAVR to assess complications such as endo leaks or calcifications [38]. However, traditional CT has limitations such as metal artifacts and blooming, which PCCT can address. van der Bie et al. found that PCCT provides high-resolution images of critical structures such as the coronary arteries and aortic valve, helping in the planning and post-surgical assessment of TAVR [38]. PCCT improves spatial resolution, reduces blooming artifacts from calcifications, and enhances calcium quantification, making it especially useful for older patients with heavy calcification [38].

Thus, the PCCT system presents numerous benefits for cardiac and coronary artery imaging, including enhanced spatial resolution, a reduction in noise and abnormalities, a lower radiation dosage, and an optimal use of contrast agents [1]. With its improved spatial resolution, this novel method potentially reduces beam hardening artifacts in patients with coronary stents and provides a more accurate evaluation of stenosis than standard cardiac and coronary CTA [1] (Table 1).

**Table 1:** Role of PCCT in clinical practice CCTA, coronary computed tomography angiography; EID-CT, energy-integrating detector computed tomography; PCCT, photon-counting computed tomography

Study	Methodology	Outcomes
Stein et al. [39]	Phantom study. Used a patient equivalent phantom scan with 12 water contrast agent filled stents of varying sized with both PCCT and EID-CT. Reconstructed images by using specific vascular kernels. Five radiologists independently evaluated the subjective image quality using a 5-point scale	PCCT-Bv56 had the highest overall reading scores. In pairwise comparison, differences were significant for PCCT-Bv56 vs. EID-CT-Bv40 for sharpness, and blooming PCCT-Bv56 with a sharp vascular kernel provided the highest diagnostic confidence and reduced the need for coronary angiograms
Petritsch et al. [40]	Phantom study. Used 28 different coronary stents of varying sizes, placed in tubes filled with contrast agent and inserted into a phantom that simulated a medium-sized patient. CT scans were taken using both the PCCT and EID-CT system. Images were analyzed to assess the visibility of the stent lumen, the increase in stent attenuation, and the level of image noise.	The differences between the two systems were statistically significant. PCCT system had better visibility of the stent lumen and lower levels of image noise. The PCCT system provided superior visibility and image quality for coronary stents compared to the traditional EID-CT system.
Si-Mohamed et al. [8]	Prospective study. 14 patients with coronary artery disease underwent CCTA with both the PCCT and EID-CT systems after contrast agent injection. The images were independently analyzed (blinded) by three radiologists.	The PCCT images had higher scores for overall quality and diagnostic confidence. The PCCT images showed greater improvement in the quality of calcifications, stents, and non-calcified plaques. The PCCT had the potential to amplify the diagnosis along with the management of coronary artery disease in clinical practice.
Van der Bie et al. [38]	A case series study using dual-source PCCT to evaluate both ex vivo heart valves and pre- and post-TAVR imaging in four patients	PCCT had some benefits compared with conventional CT such as higher spatial resolution, lower blooming artifacts of calcifications, improved delineation, and enhanced calcium quantification in both the aortic valve and coronary arteries. It led to increased accuracy in grading of coronary stenosis particularly in older people with widespread calcifications and had better description of plaque components. Accurately assessing coronary stenosis prior to TAVR can reduce the use of CTA. Utility of prosthetic valve imaging after TAVR includes improved assessment of peri-prosthetic leakage following the procedure. PCCT enhances pre- and post-TAVR planning and evaluation, providing clearer and more detailed insights.

PCCT has shown significant improvements in image quality, particularly for CCTA, plaque visualization, and stent imaging, with lower radiation doses compared to conventional methods [8,27,38,41].

Future directions and innovations

Recent advances in engineering and physics have led to the development of PCCT, which has the capability to subdue many constraints of current CT systems and significantly enhance diagnostic capabilities. Compared to traditional CT, PCCT has a number of benefits, such as lower electronic noise, enhanced spatial resolution, and enhanced material decomposition through customized energy thresholds, making it ideal for detailed imaging in cardiovascular diseases and beyond [5,21]. PCCT has shown great potential in detecting preclinical atherosclerosis, cancerous lesions, and enhancing imaging in various areas such as lung nodules, pneumonia, and chronic obstructive pulmonary disease through K-edge material decomposition and deep learning for noise reduction [40,42,43]. Its future integration with deep learning and radiomics may revolutionize medical imaging by enabling personalized diagnostics and real-time decision support [11,13,15,44]. However, further multicenter studies are required to confirm its wide clinical use.

## Conclusions

This review emphasizes the rising significance of PCCT in detecting cardiovascular illness since it provides higher resolution, less radiation, and more accuracy than traditional CT. While PCCT shows great potential for enhancing early detection and patient outcomes, challenges such as limited availability, high costs, and the need for standardized protocols remain. Addressing these issues through future research could make PCCT a transformative tool in cardiovascular care.
